# Age related medication for addiction treatment (MAT) use for opioid use disorder among Medicaid-insured patients in New York

**DOI:** 10.1186/s13011-019-0215-4

**Published:** 2019-06-25

**Authors:** Charles J. Neighbors, Sugy Choi, Shannon Healy, Rajeev Yerneni, Tong Sun, Liudmila Shapoval

**Affiliations:** 1 Center on Addiction, New York, NY USA; 2 Boston University School of Public Health, Boston, MA USA; 3 New York State Office of Alcoholism and Substance Abuse Services (OASAS), Albany, NY USA

**Keywords:** Opioid use disorder, Medication for addiction treatment, Medication-assisted treatment, MAT, Medicaid, Substance use disorder

## Abstract

**Background:**

Medication for addiction treatment (MAT) has received much attention in recent years for treating individuals with opioid use disorders (OUD). However, these medications have been significantly underused among particular subgroups. In this paper, we describe the age distribution of treatment episodes for substance use disorder among Medicaid beneficiaries in New York and corresponding MAT use.

**Methods:**

Using New York Medicaid claims, we identified individuals with OUD that received treatment for substance use disorder in 2015. The type of substance use treatment is the primary outcome measure, which includes methadone, buprenorphine, naltrexone or other non-medication treatment.

**Results:**

A total of 88,637 individuals were diagnosed with OUD and received treatment for substance use disorder and 56,926 individuals received some type of MAT in 2015, with 40.2% receiving methadone, 21.9% receiving buprenorphine and 2.2% receiving naltrexone while 21.9% received non-medication based treatment. Young adults (ages 18–29) were a large proportion (25%) of individuals in treatment for OUD yet were the least likely to receive MAT. Relative to young adults, 30–39 year olds (adjusted odds ratio [AOR] = 1.62, 95% CI = 1.56–1.68), 40–49 year olds (AOR = 1.90, 95% CI = 1.82–1.99), 50–59 year olds (AOR = 2.65, 95% CI = 2.52–2.78), and 60–64 year olds (AOR = 5.03, 95% CI = 4.62–5.48) were more likely to receive MAT.

**Conclusions:**

These preliminary findings highlight high numbers of young adults in treatment for OUD and low rates of MAT, which is not consistent with treatment guidelines. Significant differences exist in the type of medication prescribed across age. More attention is needed to address the treatment needs among individuals of different age, notably young adults.

## Background

Individuals with opioid use disorder (OUD) have high mortality, morbidity, and low remission rates [1, 2]. Medication for addiction treatment (MAT) is effective for treating OUD and multiple guidelines recommend the use of MAT—methadone, buprenorphine, naltrexone—for the treatment [3–6]. Research on MAT utilization has received much attention in recent years, but despite the robust scientific evidence and commensurate clinical guidelines recommending their use, these medications are significantly underused [7].

The opioid epidemic disproportionately affected Medicaid beneficiaries [8–10]. For instance, Medicaid beneficiaries ages 18 to 64 have a higher rate of OUD compared to privately insured individuals, comprising about 12% of all adults in this age group in 2015 [8]. Medicaid beneficiaries are more likely to experience negative health outcomes, such as overdose than those with other sources of insurance [11, 12]. Medicaid beneficiaries, especially youth, had higher rates of substance use disorders compared to privately-insured youth. Medicaid beneficiaries with an OUD have higher substance use disorder treatment rates than privately insured adults with the same condition [8].

Medicaid-covered medication treatment of OUD has increased dramatically between 2011 and 2016, from $394.2 million to $929.9 million [9]. Yet, research on MAT patterns of Medicaid beneficiaries who utilized some type of substance use disorder (SUD) treatment is limited. Existing studies of publicly funded treatment indicate low rates of MAT and even lower rates among youth [13, 14]. Since 2001, New York State has offered Medicaid coverage to low-income uninsured single adults, providing some experience that can inform other states that extend Medicaid coverage to non-elderly, childless adults [15]. The New York Medicaid program is one of the largest in the U.S. in terms of cost and total people covered and has been a large funder of SUD treatment services provided in the state [16, 17].

Overall, age is an important factor to consider when examining patterns of behavioral health and health services utilization. Although age is an important characteristic that is associated with access to substance use treatment, often, studies do not directly compare the young adults (ages 18–29) to those of other age groups. Despite the fact that young adults are undergoing a unique developmental period, there is limited understanding of the rates of evidence-based practice, OUD treatment, among young adults [18]. Since young adulthood is characterized by unique neurodevelopment and psychosocial adjustment, engaging young adults in treatment may require a different strategy than with older adults [18]. Existing studies in New York have focused on older beneficiaries [19] and young adults are an understudied population. [20, 21] Access to substance use disorder treatment services have been challenging, especially for young adults who prefer buprenorphine and naltrexone compared to methadone [22, 23], as limited access to buprenorphine providers have been an ongoing problem [24]. Age effects of the current epidemic have been understudied, especially research on MAT [25]. In this paper, we examine the use of the different types of SUD treatment (methadone, buprenorphine, naltrexone, other non-medication treatment) by age groups among patients who have at least one OUD diagnosis and at least one treatment episode for SUD in the New York State Medicaid population. We hypothesized that young New York Medicaid beneficiaries aged 18 to 29 in our sample will be less likely to engage in MAT compared to older Medicaid beneficiaries.

## Methods

Our analysis is based on New York Medicaid data for the calendar year 2015 for beneficiaries aged 18 to 64. International classification of disease diagnoses codes (ICD-10) was used to identify patients with at least one OUD diagnosis and procedure codes from Current Procedural Terminology or International Classification of Disease systems, prescription national drug codes, or from New York Medicaid specific reimbursement codes indicating SUD treatment were used to identify patients who received at least one treatment for SUD in 2015. Patients with health insurance coverage through both Medicaid and Medicare were excluded because we were unable to access the entirety of their healthcare claims data.

The primary outcome of interest was the type of SUD treatment utilization that patients received in 2015. Patients were classified as having received methadone, buprenorphine or naltrexone treatment if they were identified with at least one methadone maintenance therapy visit, or if they filled at least one buprenorphine or naltrexone-based prescription. Among those who did not receive MAT, we defined SUD treatment based on billing procedure codes from Current Procedural Terminology or ICD-10, or from New York Medicaid specific reimbursement codes indicating SUD treatment. Treatment included inpatient, outpatient, or psychotherapy care with a SUD diagnosis; yet, it excluded detoxification services if not followed by specific treatment for substance use disorder. We created four mutually exclusive groups: methadone, buprenorphine, naltrexone and other treatment. For those patients who used multiple medications, we assigned them to the category of their most frequently used medication.

The following six sociodemographic variables, including age group (18–29, 30–39, 40–49, 50–59, 60–64 years), place of residence (New York City (NYC), outside NYC), race and ethnicity (non-Hispanic white, black, Latinx, other, unknown), sex (female, male), and Medicaid eligibility months were considered in the analysis.

Treatment utilization was graphically displayed by age group to help highlight age patterns in the data. The x-axis represents age groups and the y-axis indicates the percentage of patients or number of patients who utilized methadone, buprenorphine, naltrexone, or other SUD treatment without medication treatment. Descriptive statistics were performed by age group. We conducted two sample z-test and t-test to compare the outcome measures and covariates across each age group. We estimated multivariable logistic regression models to identify age group differences in OUD MAT utilization after adjusting for individual covariates. All statistical analyses were conducted using SAS 9.4 and STATA M.P.13.

## Results

**Fig. 1:**
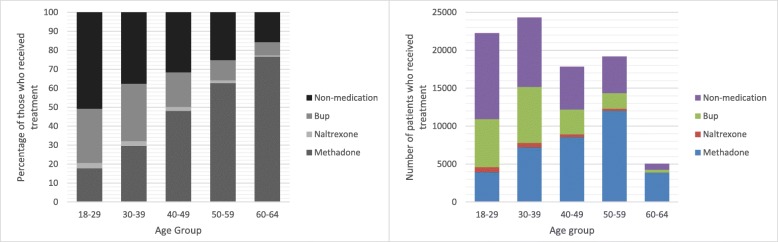
Opioid use disorder (OUD) medication treatment types among OUD patients by age distribution

In total, 88,637 individuals aged 18–64 received at least one treatment for SUD among 111,033 individuals who had at least one OUD diagnosis in 2015. Figure 1 shows that young adults (ages 18–29) highlight a large proportion of beneficiaries who have received treatment for OUD (25% of all individuals in treatment for OUD), yet only 49.2% were receiving MAT compared to 62.4% of 30 to 39 year olds who received MAT. Overall, 64.2% of all individuals in treatment for OUD received some type of MAT in 2015, with 40.2% receiving methadone, 21.9% receiving buprenorphine and 2.2% receiving naltrexone while 35.8% received non-medication based treatments. Of those receiving non-medication based treatment, the majority of patients (81.5%) received outpatient behavioral care services, while 5.1% only had inpatient care, and 13.4% received a combination of inpatient and outpatient services during the year.

Demographic characteristics varied by age for this sample (Table 1). Young adults (18–29 years old) are less racially diverse than older age groups, with nearly 73.3% identifying as non-Hispanic white, compared to 64.6% of 30–39 year olds, 35% of 40–49 year olds, 20.9% of 50–59 year olds, and 20.2% of 60–64 year olds. Young adults had greater proportions of females (39.9%) compared to only 38.8, 32.4, 32.3, and 31.9% of 30–39, 40–49, 50–59, and 60–64 year olds, respectively. Additionally, 79.8% of young adults reside outside of New York City compared to 69.5% of 30–39 year olds, 40.7% of 40–49 year olds, 25% of 50–59 year olds, and only 20.3% of 60–64 year olds. The mean length of Medicaid eligibility months was 10.6 months for young adults compared to 10.9 months for 30–39 year olds, 11.1 months for 40–49 year olds, 11.4 months for 50–59 year olds, and 11.5 months for 60–64 year olds, respectively.

**Table 1:** Demographics characteristics and Medication-assisted treatment (MAT) use by age groups

	*Age*					
	18–29 *n* = 22,270	30–39 *n* = 24,314	40–49 *n* = 17,819	50–59 *n* = 19,184	60–64 *n* = 5050	Total *n* = 88,637
*Race n (%)*						
Non-Hispanic White	16,327 (73.3)	15,697*** (64.6)	6237*** (35.0)	4001*** (20.9)	1019*** (20.2)	43,281 (48.8)
Non-Hispanic Black	1577 (7.1)	1951*** (8.02)	3074*** (17.3)	5475*** (28.5)	1696*** (33.6)	13,773 (15.5)
Latinx	1688 (7.6)	3792*** (15.6)	5830*** (32.7)	6926*** (36.1)	1674*** (33.2)	19,910 (22.5)
Other	1282 (5.8)	1894*** (7.8)	2107*** (11.8)	2366*** (12.3)	586*** (11.6)	8235 (9.3)
Unknown	1396 (6.3)	980*** (4.0)	571*** (3.2)	416*** (2.2)	75*** (1.5)	3438 (3.9)
*Gender n (%)*						
Female	8874 (39.9)	9429* (38.8)	5766*** (32.4)	6199*** (32.3)	1611*** (31.9)	31,879 (36.0)
Male	13,396 (60.2)	14,885** (61.2)	12,053*** (67.6)	12,985*** (67.7)	3439*** (68.1)	56,758 (64.0)
*Location n (%)*						
New York City (NYC)	4502 (20.2)	7428*** (30.6)	10,562*** (59.3)	14,385*** (35.0)	4023*** (80.0)	40,900 (46.1)
Outside NYC	17,768 (79.8)	16,886*** (69.5)	7257*** (40.7)	4799*** (25.0)	1027*** (20.3)	47,737 (53.9)
*Mean Medicaid eligibility months, (standard deviation)*	10.6 (2.5)	10.9*** (2.3)	11.1*** (2.1)	11.4*** (1.8)	11.5*** (1.6)	11.0 (2.2)
*Treatment Utilization n (%)*						
Medication-Assisted Treatment (MAT)	10,948 (49.2)	15,172*** (62.4)	12,181*** (68.4)	14,363*** (74.9)	4262*** (84.4)	56,926 (64.2)
-Methadone	3950 (17.7)	7209*** (29.7)	8554*** (48.0)	12,034*** (62.7)	3868*** (76.6)	35,615 (40.2)
-Naltrexone	656 (3.0)	584*** (2.4)	374*** (2.1)	278*** (1.5)	37*** (0.7)	1929 (2.2)
-Buprenorphine	6342 (28.5)	7379*** (30.4)	3253*** (18.3)	2051*** (10.7)	357*** (7.1)	19,382 (21.9)
Non-Medication Treatment	11,322 (50.8)	9142*** (37.6)	5638*** (31.6)	4821*** (25.1)	788*** (15.6)	31,711 (35.8)

*P-value calculated from two sample z-test and t-test to compare patients in each age group with 18–29 year olds. *p < 0.05, **p < 0.01, ***p < 0.001

The percentages of patients who received MAT were significantly larger in older patients compared with young adults. Older age groups had a higher prevalence of MAT than younger groups with 84.4% of those aged 60–64 receiving some type of medication for addiction treatment compared to 74.9% of those aged 50–59, 68.4% of those aged 40–49, 62.4% of those aged 30–39 and 49.2% of young adults aged 18–29. In adjusted comparisons (Table 2), older patients, 30–39 year olds (AOR = 1.62, 95% CI = 1.56–1.68), 40–49 year olds (AOR = 1.90, 95% CI = 1.82–1.99), 50–59 year olds (AOR = 2.65, 95% CI = 2.52–2.78), and 60–64 year olds (AOR = 5.03, 95% CI = 4.62–5.48) were more likely to receive medication treatment for OUD compared to young adults. Latinx (AOR = 1.08, 95% CI = 1.03–1.14) and beneficiaries living in NYC (AOR = 2.29, 95% CI = 2.21–2.38) were more likely to receive MAT compared to Non-Hispanic whites and beneficiaries living outside NYC, respectively. Non-Hispanic blacks (AOR = 0.36, 95% CI = 0.34–0.38) and females (AOR = 0.73, 95% CI = 0.71–0.75) were less likely to receive MAT compared to Non-Hispanic whites and females.

**Table 2:** Multivariable regression analyses of the relationship between age group and receipt of MAT

	*Crude Odds Ratio*	*95% Confidence Interval*	*Adjusted Odds Ratio*	*95% Confidence Interval*
*Age group*				
18–29	Reference		Reference	
30–39	1.72***	1.65–1.78	1.62***	1.56–1.68
40–49	2.23***	2.14–2.33	1.90***	1.82–1.99
50–59	3.08***	2.95–3.21	2.65***	2.52–2.78
60–64	5.59***	5.16–6.06	5.03***	4.62–5.48
*Race n*				
Non-Hispanic White	Reference		Reference	
Non-Hispanic Black	0.82***	0.79–0.85	0.36***	0.34–0.38
Latinx	2.39***	2.30–2.49	1.08***	1.03–1.14
Other	1.40***	1.33–1.48	0.67***	0.63–0.71
Unknown	0.63***	0.59–0.68	0.52***	0.48–0.56
*Gender*				
Female	Reference		Reference	
Male	0.83***	0.81–0.86	0.73***	0.71–0.75
*Location*				
Outside NYC	Reference		Reference	
NYC	2.48***	2.41–2.55	2.29***	2.21–2.38

*p < 0.05, **p < 0.01, ***p < 0.001

## Discussion

This study examines MAT utilization by age groups among Medicaid beneficiaries identified with OUD who received at least some type of SUD treatment in 2015. Our results demonstrate that young adults are a large proportion of Medicaid beneficiaries in treatment for OUD yet are less likely to receive MAT. Notably, we found the use of MAT among the Medicaid beneficiaries with OUD is higher compared to what has been previously reported by the National Surveys on Drug Use and Health [26]. This alludes that Medicaid coverage may facilitate access to SUD treatment yet highlights the need for extra attention to young adults.

Only half of young adults received MAT for OUD, and the older patients were more likely to receive MAT. A recent study of commercial claims data from 2001 to 2014 found that only 25% of youth (adolescents and young adults, ages 13–25) with OUD received any form of medications for OUD [27]. The low rates of MAT among young adults who received treatment for SUD is concerning as previous studies have shown the efficacy of MAT and less likelihood of relapse among young adults [14, 27]. The low proportion of individuals receiving MAT is not concordant with standard treatment guidelines [3, 6]. The findings highlight a need to understand why MAT is less likely to be used as a treatment for OUD among young adults compared to other age groups and address this gap in care practices.

Medication utilization differed across the age groups, with relatively high use of buprenorphine among young adults and relatively high use of methadone among older patients. This may be due to the differences in patient or provider attitudes toward MAT where young adults favoring buprenorphine compared to methadone [28, 29]. The higher rates of methadone use among older adults have been previously reported and may reflect older individuals entering treatment before buprenorphine became available or who having greater clinical case complexity for which methadone programs are better suited [30]. Previous studies document the importance of community attitudes about the appropriateness of medications for youth, many patients and treatment providers have been reluctant to use medications in early episodes of care for OUD [29, 31, 32].

Further research is needed on the types and quality of treatment available to youth and young adults with OUD [18, 33, 34]. Brain development extends into the mid 20’s, which means that youth are passing through a heightened period of vulnerability from exposure to substances [35, 36]. Not only is this period a time of increased risk of developing substance use dependence but also a time when prolonged exposure to substances can have lasting effects on brain development [35]. This developmental transition presents a dilemma when there are also concerns that the MAT can also create lasting changes in the brain [34]. Because of these concerns and because of community attitudes about the appropriateness of medications for youth, many patients and treatment providers have been reluctant to use medications in early episodes of care for OUD [29, 31, 32].

Often when MAT is incorporated into care, treatment professionals and patients/family members will use medications for a brief transition period with the expectation that the youth quickly move into medication-free psychosocial treatment to maintain recovery. However, two studies have found that rapid tapering of youth off medications leads to a heightened risk of relapse [37, 38]. More research is needed to investigate the appropriate lengths of MAT among young adults. Generally, a briefer MAT treatment duration is associated with poor outcomes [39–42]. Yet, when medications are incorporated into treatment, younger individuals are less compliant and more likely to quit treatment than older individuals [43, 44]. All of this raises critical questions about the risk/benefits of longer-term treatment with medications for youth. Both treatment providers and patients/family members need more evidence about the relative effectiveness of MAT for OUD for treatment planning during initial phases of care [18, 33].

This study has several limitations. This is a cross-sectional analysis of data without individual clinical factors, such as the severity of OUD, so findings indicate associations rather than cause and effect relationships. Our sample has been restricted to those who have OUD diagnosis, but not all individuals who receive treatment for OUD have a formal OUD diagnosis [45]. The data only captures treatment services that have been billed through Medicaid in New York. While New York has a large, diverse population and treatment system for substance use disorders funded through its Medicaid program, the findings may not mirror patterns of care in other regions. Finally, our use of Medicaid data to document MAT utilization for substance use disorders does not cover potential treatment events that were not reimbursed by Medicaid. However, the individuals in this study are identified with OUD to Medicaid, which covers the large proportion of individuals with SUD in the United States and is an important component of healthcare reform [46].

## Conclusions

We found large numbers of young adults seeking treatment for OUD, low use of MAT, and differences in medication use by age. Future research is needed to identify how to increase the uptake of MAT while attending to specific needs across different age groups. Especially, more research is needed for engaging young adults who have disproportionately low rates of using MAT after engaging in SUD treatment. Public health officials and treatment providers should consider age group effects in designing individually tailored interventions for patients for engagement with medications in the course of their OUD treatment.

## Data Availability

The data are provided by the New York Department of Health, who have strong safeguards and access restrictions to protect individual privacy. The New York Department of Health did not participate in preparation of this manuscript. The content is solely the responsibility of the authors and does not necessarily represent the official views of the New York Department of Health.

## Abbreviations

ICD-10: International classification of disease diagnoses codes
MAT: Medication for addiction treatment
NYC: New York City
OUD: Opioid use disorder
SUD: Substance use disorder
